# Detoxification of Insect-Derived Allergen PLA2 via Quercetin Modification: Molecular Simulation and Animal Validation

**DOI:** 10.3390/nu17172872

**Published:** 2025-09-04

**Authors:** Fukai Li, Liming Wu, Min Wang, Enning Zhou, Fei Pan, Jian Zhou, Mengrui Yang, Tongtong Wang, Liang Li, Qiangqiang Li

**Affiliations:** 1 Institute of Quality Standard and Testing Technology for Agro-Products, Chinese Academy of Agricultural Sciences (CAAS), Beijing 100081, China; fk_liharrison@163.com (F.L.); zhoujian_8382@163.com (J.Z.); yangmengrui2014@163.com (M.Y.); wangttong123@126.com (T.W.); liliang@caas.cn (L.L.); 2 State Key Laboratory of Resource Insects, Institute of Apicultural Research, Chinese Academy of Agricultural Sciences (CAAS), Beijing 100093, China; zhouenning0425@163.com (E.Z.); yunitcon@yeah.net (F.P.); 3 Hainan Academy of Agricultural Sciences, Haikou 571100, China

**Keywords:** insect protein, allergen, phospholipases A2, quercetin, mouse model

## Abstract

Background: Insect-derived proteins constitute an underutilized biological resource requiring urgent exploration to address global food protein shortages. However, their widespread application is hindered by the allergenic potential, particularly phospholipase A2 (PLA2), a highly immunoreactive allergen prevalent in edible insects such as ants and honeybees. Objective: This study systematically investigated the molecular mechanism underlying quercetin-mediated reduction in PLA2 allergenicity, aiming to establish a novel strategy for developing hypoallergenic insect protein resources. Methods and Results: Through integrated computational and experimental approaches, we identified quercetin’s dual non-covalent and covalent binding capabilities with PLA2. Molecular docking revealed robust interactions (the binding energy of −6.49 kcal/mol) within the catalytic pocket. Meanwhile, mass spectrometry specifically identified Cys37 as the covalent modification site, which can bind to quercetin and increase the gyration radius (Rg) of PLA2 within 75–125 ns. Molecular dynamics simulations illustrated quercetin-induced conformational changes affecting critical antigenic epitopes. Murine experiments further confirmed that quercetin-modified PLA2 exhibited significantly reduced IgE reactivity and allergic responses compared to native PLA2, as demonstrated by assessments of anaphylactic behavior, histopathological changes, and measurements of serum IgE antibody and biogenic amine levels. Conclusions: Collectively, these findings provide a transformative approach to safely utilize insect-derived proteins for sustainable nutrition solutions.

## 1. Introduction

The exponential growth of the population (which is expected to reach 10 billion by 2050) has brought about the social challenges of ensuring their demand for protein foods [1]. Nowadays, researchers are considering insects as a potential source of alternative proteins due to their low environmental impact and high protein content (30–65%) [2]. Approximately 2000 edible insect species at various life stages were consumed globally in 2017, with ants, wasps, and bees (Hymenoptera, 15%) ranking highest [3,4]. However, their production of highly allergenic phospholipase A2 (PLA2) triggers anaphylaxis in 57–97% of allergic patients [5,6]. Furthermore, PLA2 combined with multivalent cations exhibits increased hemolytic activity, enabling rapid bloodstream entry and subsequent damage to organs [7,8]. In recent years, research on PLA2 has mostly focused on detection [9,10,11,12,13], with only a few studies aimed at reducing allergens, which also poses a challenge to the widespread application of alternative proteins. Therefore, effectively reducing PLA2’s allergenicity holds paramount importance for global health and expanding the utilization of insect-derived proteins resources.

Polyphenols possess the capacity to engage in both noncovalent and covalent interactions with proteins, leading to alterations in protein structure and potential masking or destruction of specific antigenic epitopes. Consequently, these interactions may contribute to a reduction in allergenicity/immunogenicity of the proteins [14,15]. Previous investigations have documented the interaction between polyphenols and diverse allergens, for instance, between peanut allergen and epigallocatechin-3-gallate [16], soybean allergen and gallic acid [17], beta-lactoglobulin and caffeic acid [18], ovalbumin and chlorogenic acid [19], wheat gliadin and luteolin [20], as well as our study about bee pollen allergen and quercetin [21]. Hence, it is plausible that the allergenic properties of food allergens could be modified through noncovalent and covalent interactions between polyphenols and allergens.

Accordingly, polyphenols can interact with allergens through non-covalent forces, including van der Waals interactions, hydrogen bonding, and hydrophobic interactions, to mitigate their allergenicity. But, the resulting complex formed in this manner is inherently unstable and susceptible to dissociation under external influences [22]. Alternatively, polyphenols can form stable complexes with allergens through covalent bonds via enzymatic reactions, free radical actions, or an alkaline environment, leading to a more effective reduction in allergenicity [23]. However, due to variations in structural characteristics among different kinds of allergens, their interactions with polyphenols are likely to vary. Molecular docking is a key in silico screening technique, widely utilized for analyzing, designing, or predicting ligand-receptor interactions, such as those between polyphenols and proteins [24]. Currently, limited knowledge exists regarding the impact and mechanism of interaction between polyphenols and PLA2. This lack of understanding introduces uncertainty concerning the further development of insect-derived proteins resources.

To address this critical challenge, we present a systematic investigation on quercetin-mediated structural reprogramming of insect-derived PLA2 for allergenicity mitigation. Our study integrates multi-scale computational approaches (molecular docking and all-atom molecular dynamics simulations) with high-resolution mass spectrometry to elucidate the molecular determinants of PLA2’s allergenicity attenuation through QR modification. This multi-level strategy enables: (1) atomic-level visualization of QR-PLA2 interaction patterns, (2) precise identification of covalent conjugation sites, and (3) quantitative assessment of epitope landscape alterations. This study not only reveals the primary masking mechanism of QR through a molecular dynamics simulation system, but also verifies the feasibility of the prepared method using a murine sensitization model, which provides a reference for the detoxification of insect-derived protein allergens in the future.

## 2. Materials and Methods

### 2.1. Reagents

HPLC-grade methanol, acetonitrile, and formic acid were sourced from Fisher Scientific (Thermo Fisher Scientific, Waltham, MA, USA). Ultrapure water was purified using a Millipore Milli-Q system (Milli-Q, Billerica, MA, USA). Sequence-grade trypsin came from Promega (Promega, Madison, WI, USA). Quercetin (HPLC grade, ≥98% purity) and honeybee (*Apis mellifera*)-derived PLA2 were acquired from Yuanye (Shanghai, China). The human native IgE antibody (ab65866) and HRP-conjugated anti-human IgE mouse monoclonal antibody (ab99806) were supplied by Abcam Inc. (Cambridge, UK).

### 2.2. Molecular Docking Analysis

Molecular docking preceded polyphenol selection for PLA2 covalent binding. The PLA2 3D structure (PDB ID: 1POC) was acquired from RCSB PDB and preprocessed by removing water molecules and adding hydrogen bonds and Gasteiger charges using Autodock Tools 1.5.7. Ligand 2D structures—chlorogenic acid (CA, CID: 1794427), curcumin (CC, CID: 969516), epigallocatechin gallate (EGCG, CID: 65064), gallic acid (GA, CID: 370), and quercetin (QR, CID: 5280343)—were downloaded from PubChem and converted to 3D formats via ChemDraw 22. Ligands underwent MM2 optimization and were docked using the Lamarckian genetic algorithm (LGA), with 10 GA runs per compound in a 126 × 126 × 126 Å^3^ grid (spacing 0.375 Å; center X = 42.016, Y = 31.811, Z = 27.098). Complexes exhibiting the lowest binding energy underwent interaction analysis with Autodock Tools 1.5.7 and visualization in PyMOL 1.8.

### 2.3. Pretreatment of PLA2 with QR

PLA2 was modified with QR via free radical grafting [25]. The grafting solution contained vitamin C (0.03 M) and H_2_O_2_ (0.1 M) dissolved in ultrapure water. PLA2 was dissolved in ultrapure water (1.0 mg/mL), then mixed with QR (0.1 mg/mL) and radical solution (120 μL). The mixture was exposed to air for incubation (RT, 12 h). Finally, the PLA2 covalent complex was ultrafiltered using a 10 kDa Millipore filter (Burlington, MA, USA) for analysis.

### 2.4. Identification of the PLA2 and PLA2-QR Conjugate by Easy-nLC1000-LTQ-Orbitrap Elite MS

Protein pretreatment followed a published method [26]. Protein solution was combined with NH_4_HCO_3_ and dithiothreitol (DTT), incubated (RT, 1 h), then alkylated using iodoacetamide (IAA). The acetylated protein was digested into peptides using sequence-grade trypsin (37 °C, 12 h). The reaction was quenched with 1 μL formic acid. Peptides were purified/enriched using a Millipore Ziptip C18 column (Burlington, MA, USA), and concentration measured via Nanodrop2000 (Thermo Fisher Scientific, USA). After concentration normalization, samples underwent instrument analysis (Thermo Fisher Scientific, USA). Conditions matched prior work [26]; Xcalibur 2.2 software (Thermo Fisher Scientific, USA) collected raw data, and Peaks DB 8.5 software (Bioinformatics Solutions Inc., Waterlo, ON, Canada) identified peptides.

### 2.5. Molecular Dynamics Simulation Analysis

Molecular dynamics simulations of PLA2 and PLA2-QR conjugate were conducted for 500 ns per system using GROMACS 21.4 [27], totaling 1.0 μs across two systems. The Amber14sb_parmbsc1 force field with explicit solvation was applied [28]. Each system was solvated in a TIP3P water box, neutralized with counterions. Energy minimization proceeded via steepest descent (1000.0 kJ/mol/nm) followed by conjugate gradient optimization (100.0 kJ/mol/nm). Subsequent pre-equilibration included NVT (0.5 ns) and NPT (0.5 ns) ensembles. Production simulations (500 ns/system) utilized an RTX 3090 GPU (NIVIDA, Santa Clara, CA, USA). Unspecified parameters followed prior methods [29]. RMSD, RMSF, SASA, and Rg were computed using gmx rms, gmx rmsf, gmx sasa, and gmx gyrate, respectively. Structures were visualized in VMD 1.9.4 software [30].

### 2.6. Animal Experiment

Mouse experiments took place at the Beijing Animal Experimental Center, approved by the IAR-CAAS Animal Ethics Committee. Thirty-two female BALB/c mice (6 weeks, 16–20 g) were housed in SPF-standard facilities (20–24 °C, 50 ± 5% humidity) with ad libitum AIN-93 diet, sterile water, and filtered air under 12/12 h light/dark. After one-week adaptation, mice were randomly divided into four groups (*n* = 8): untreated control, OVA (positive control), PLA2, and PLA2-QR conjugate. Over 28 d, the control received weekly i.p. saline; the OVA group received weekly i.p. 0.2 mL OVA (0.1 mg/mL with 1% alum) for three weeks, then 0.2 mL OVA (0.5 mg/mL with 1% alum) on day 28. PLA2 and PLA2-QR groups received equivalent protein concentrations. Post-final injection, anaphylactic behavior was monitored for 15 min. Mice were then euthanized via gradual CO_2_ displacement (10–30%/min) in an IVC cage until unconsciousness and respiratory arrest. Orbital blood was collected, clotted naturally for 1 h, and serum isolated (3000× *g*, 10 min) for IgE and bioamine analysis.

### 2.7. Anaphylactic Behavior Monitoring and Histopathological Test

Post-final injection, mice were observed for 15 min to assess anaphylaxis (index scored based on scratching/sneezing frequency). For histopathology, formalin-fixed spleen tissue was paraffin-embedded, sectioned, and stained separately with hematoxylin-eosin (HE) and toluidine blue (TB). Sections were examined using a Nikon Eclipse Ci microscope (Nikon, Tokyo, Japan).

### 2.8. Detection of IgE Antibody and Bioamines

Serum IgE antibody levels were measured via ELISA kit (CLOUD-CLONE CORP., Houston, TX, USA). Bioamine concentrations were analyzed by an Agilent UPLC system with a C18 column. Serum preparation followed Tao et al. [31]. Column conditions: 40 °C, 0.3 mL/min flow rate. Mobile phase A: 0.1% (*v*/*v*) formic acid + 2 mM ammonium formate in water; phase B: methanol. Gradient elution and MS parameters for histamine, octopamine, and tryptamine appear in Tables S1–S3 (Supplementary Materials).

### 2.9. Statistics

Treatment group differences were analyzed via one-way ANOVA using SPSS 26.0. Post hoc multiple comparisons were performed with Tukey’s test when ANOVA indicated significant effects (*p* < 0.05).

## 3. Results and Discussion

### 3.1. Molecular Docking Analysis of Five Polyphenols Bound to PLA2

**Figure 1:**
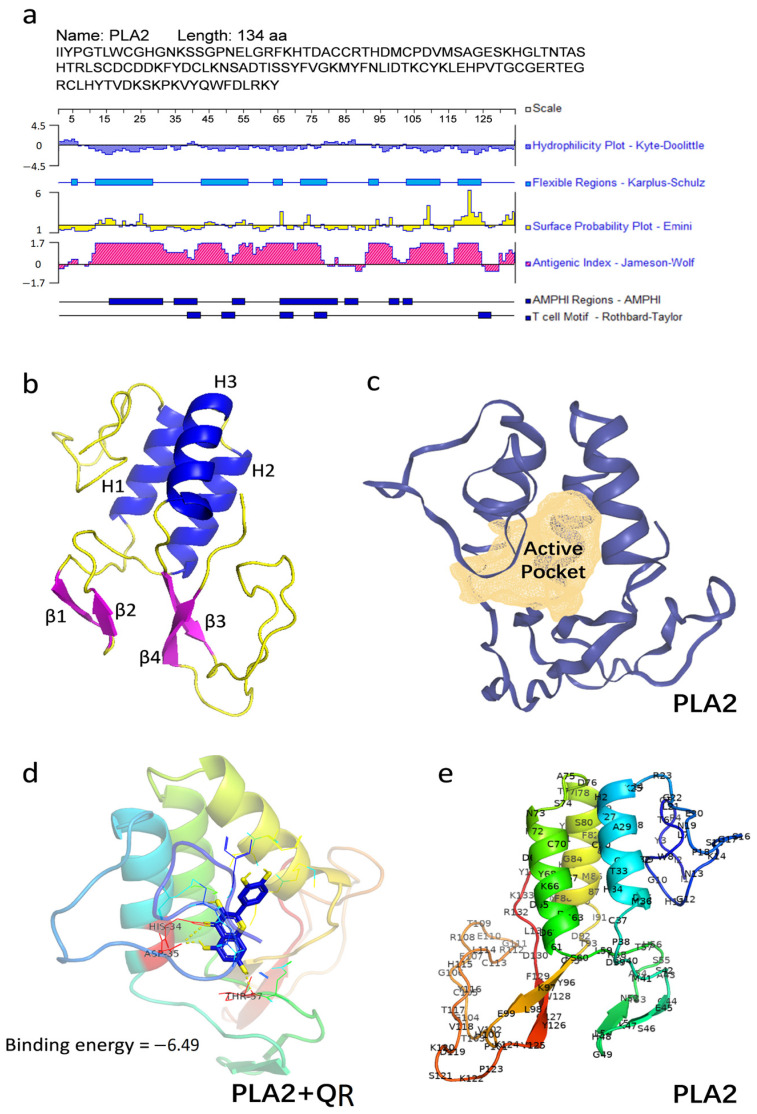
(**a**) The hydrophilicity, surface probability, and flexible regions, as well as the B and T cell epitopes of PLA2 analyzed by DNAStar software. (**b**) The 3D structure model of PLA2 visualized by PyMOL software. (**c**) A diagram showing the active pocket of PLA2. (**d**) Molecular docking results of quercetin (QR) binding with PLA2. (**e**) A 3D model depicting the spatial distribution of amino acid residues in PLA2. PLA2: Phospholipase A2, an allergen from honeybee (*Apis mellifera*).

We selected chlorogenic acid (CA), curcumin (CC), epigallocatechin gallate (EGCG), gallic acid (GA), and quercetin (QR) that have been previously reported to potentially reduce allergenicity as candidates for molecular docking simulation with PLA2. We aimed to identify the polyphenol with the highest affinity towards PLA2. Initially, we analyzed the structural properties and antigenic regions of PLA2 using DNAStar 11.1 software and PyMOL software. PLA2 features three α-helices (H1-3) and four β-sheets (β1-4) (Figure 1a,b). Antigenic epitopes primarily align with hydrophobic/flexible regions (residues 10–80, 90–125). Over 75% epitope coverage confirms strong immunogenicity. Molecular docking predicted binding energies/conformations of five polyphenols with PLA2 (Table S4):

- CA: HIS34, ASP35, GLY10 (−4.98 kcal/mol)
- CC: HIS34, ASP35 (−5.83 kcal/mol)
- EGCG: HIS34, ASP35, GLY10 (−6.42 kcal/mol)
- GA: ILE1, HIS11, ASN13 (−3.67 kcal/mol)
- QR: HIS34, ASP35, THR57 (−6.49 kcal/mol)

As established in the literature, a negative binding energy signifies a spontaneous non-covalent interaction between ligands and receptors, with more negative values corresponding to stronger binding affinity [32]. Previous studies report stable affinities for related systems: β-casein bound to chrysin, apigenin, and luteolin with binding energies of −7.11, −7.18, and −7.31 kcal/mol, respectively [33]. Similarly, bovine serum albumin (BSA) exhibited stable binding to EGCG, ECG, EGC, and EC with binding energies of −8.24, −9.02, −6.92, and −7.29 kcal/mol, respectively [34]. Comparatively, in our study, QR exhibited the lowest binding energy (−6.49 kcal/mol) to PLA2, indicating a stable binding affinity. This stable interaction is further visualized by the molecular docking conformation (Figure 1d) within the PLA2 active pocket (Figure 1c), demonstrating a complementary lock-and-key binding mode.

### 3.2. The Identification of PLA2 and PLA2-QR Conjugate and Its Binding Site

**Figure 2:**
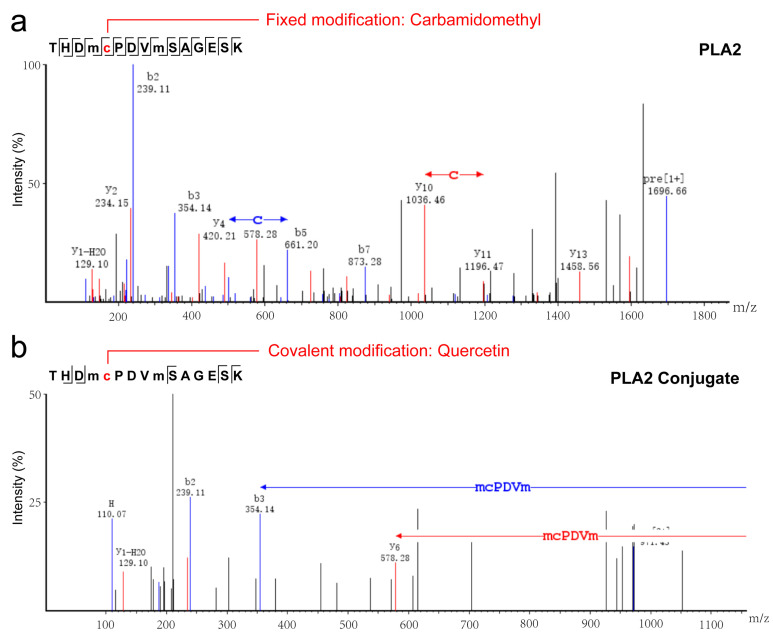
The peptide analysis of (**a**) PLA2 and (**b**) PLA2-QR conjugate digested by sequence-grade trypsin via Easy-NLC1000-LTQ-Orbitrap Elite Mass Spectrometer. PLA2: Phospholipase A2, an allergen from honeybee (*Apis mellifera*).

Following the interaction of QR with PLA2 during conjugate formation, both PLA2 and its conjugate were identified after trypsinization. As shown in Table 1, both PLA2 and PLA2-QR conjugate shared ten peptide fragments, except the fragment THDMCPDVMSAGESK which exhibited significant differences. One QR molecule could covalently modify the Cys37 residue in the THDMCPDVMSAGESK fragment of PLA2 after treatment (Figure 2a,b). In the free-radical grafting method, a redox pair of hydrogen peroxide and ascorbic acid is commonly utilized to generate hydroxyl radicals to oxidize amino acids on protein side chains. Ultimately, the oxidized protein can react with polyphenols to form cross-linked conjugate [23]. In our investigation, Cys37 on PLA2 could be oxidized by hydroxyl radicals in the radical reaction system, leading to the formation of a covalent complex between PLA2 and QR through binding the oxidized Cys37 residue. Furthermore, as reported in our previous study [13], the key antigenic epitopes of PLA2 are located at residues AA26–40 (HTDACCRTHDMCPDV), AA55–72 (SHTRLSCDCDDKFYDCLK), AA76–85 (DTISSYFVGK), AA91–97 (IDTKCYK), and AA110–127 (EGRCLHYTVDKSKPKVYQ). Cys37 is situated within the antigenic epitopes of PLA2 and located at the α-helix/random coil junction (Figure 1a,e), suggesting that covalent modification may alter conformational epitopes and modify PLA2 allergenicity. The above results further reveal the mechanism of Cys37 covalent binding for QR-induced epitope masking, and also establish a technical framework for developing the next generation of hypoallergenic insect protein components.

### 3.3. Molecular Dynamics Simulation of PLA2 and PLA2-QR Conjugate

**Figure 3:**
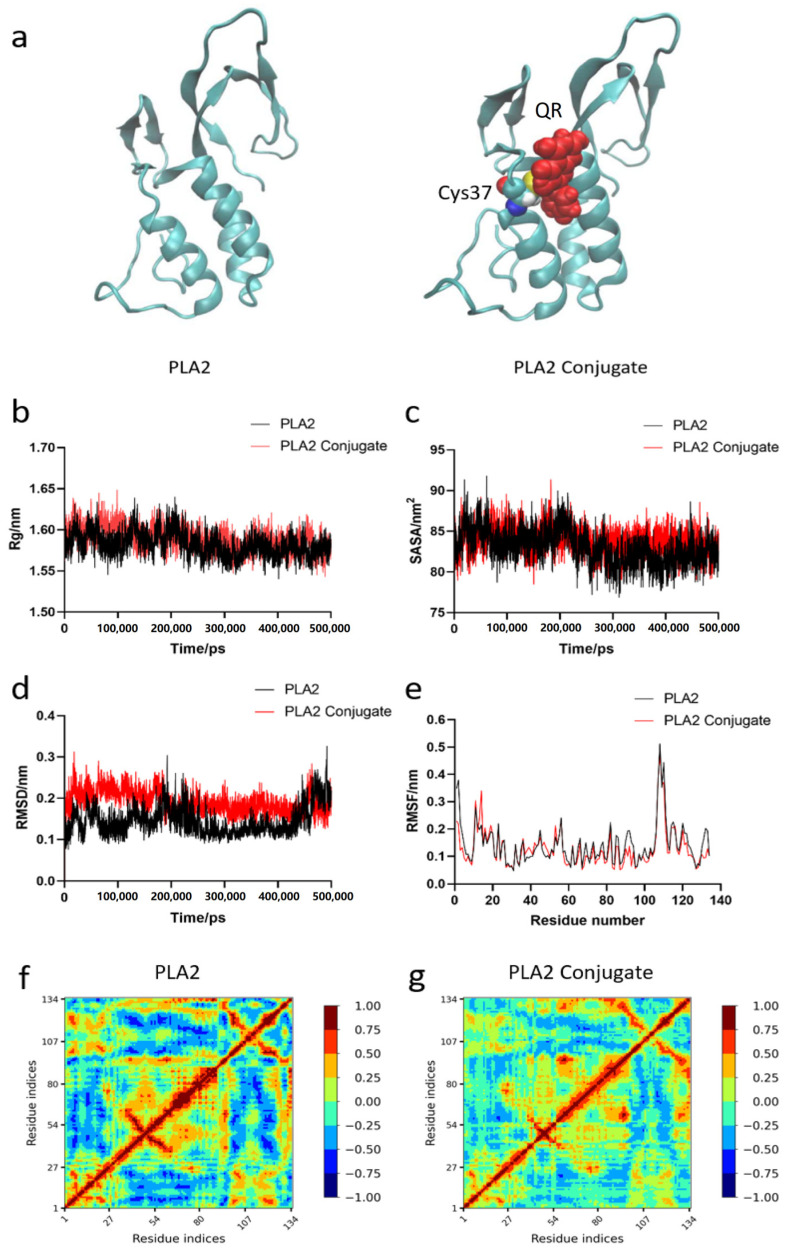
(**a**) The molecular simulation of PLA2 and PLA2-QR conjugate. (**b**) The gyration radius (Rg), (**c**) solvent accessible surface area (SASA), (**d**) root-mean-square deviation (RMSD), and (**e**) root mean square fluctuation (RMSF) analysis plots of PLA2 and PLA2 conjugate. The dynamical cross-correlation matrix (DCCM) heat map of (**f**) PLA2 and (**g**) PLA2 conjugate. PLA2: Phospholipase A2, an allergen from honeybee *(Apis mellifera*).

Molecular dynamics simulation enables the investigation of structural changes and the molecular mechanisms underlying protein-ligand interactions under specific conditions [35]. Therefore, we employed molecular dynamics simulations to explore the dynamic structural changes associated with PLA2-QR covalent conjugation (see Videos S1 and S2). The gyration radius (Rg) indicates the relative motion of PLA2 atoms before and after QR modification [36]. As shown in Figure 3a,b, QR covalently binds to Cys37 of PLA2, leading to an increased Rg value of PLA2 within 75–125 ns of simulation. This indicates enhanced molecular motion and conformational stretching of PLA2 during this period. However, with the continuous action of QR, the molecular conformation of PLA2 tends to stabilize. The solvent-accessible surface area (SASA) can reflect the interaction between PLA2 and solvents pre- and post-modification [35]. As shown in Figure 3c, the average SASA value of PLA2 in the 0–500 ns timeframe was 83.07, while the average SASA value of the modified PLA2-QR conjugate was 83.84 within the same timeframe, indicating that QR modification resulted in a reduction in hydrophobicity for PLA2. The root-mean-square deviation (RMSD) measures PLA2 stability pre/post modification, while RMSF indicates atomic fluctuation amplitudes in PLA2 and its conjugate [37]. We found that the RMSD value of PLA2 significantly increased after covalent modification, indicating a steady decrease in systemic stability (Figure 3d). However, we found no significant variation amplitude in PLA2 pre- and post-modification (Figure 3e). The dynamic cross-correlation matrix (DCCM) can be used to analyze the motion correlation between specific atoms of each amino acid in PLA2 pre- and post-modification [38]. The DCCM heat map uses red to indicate consistent directional movement and blue for opposing motion in the region, and the color depth reflects the intensity of the movement. Compared to PLA2, the motion correlation in β1 (44–47), β2 (50–53), and the H2 (61–73) regions was weaker in the PLA2-QR conjugate than in PLA2 (Figure 3f,g), indicating structural motion changes caused by QR covalent modification. In summary, the covalent modification of QR weakened the motion correlation among β1, β2, and H2 structures, while decreasing the systemic stability of PLA2 and therefore increasing its solvent affinity. QR conjugation-induced structural changes in PLA2 may mask or destroy its antigenic epitopes [39].

### 3.4. The Allergenicity Assessment of PLA2 and PLA2-QR Conjugate In Vivo

**Figure 4:**
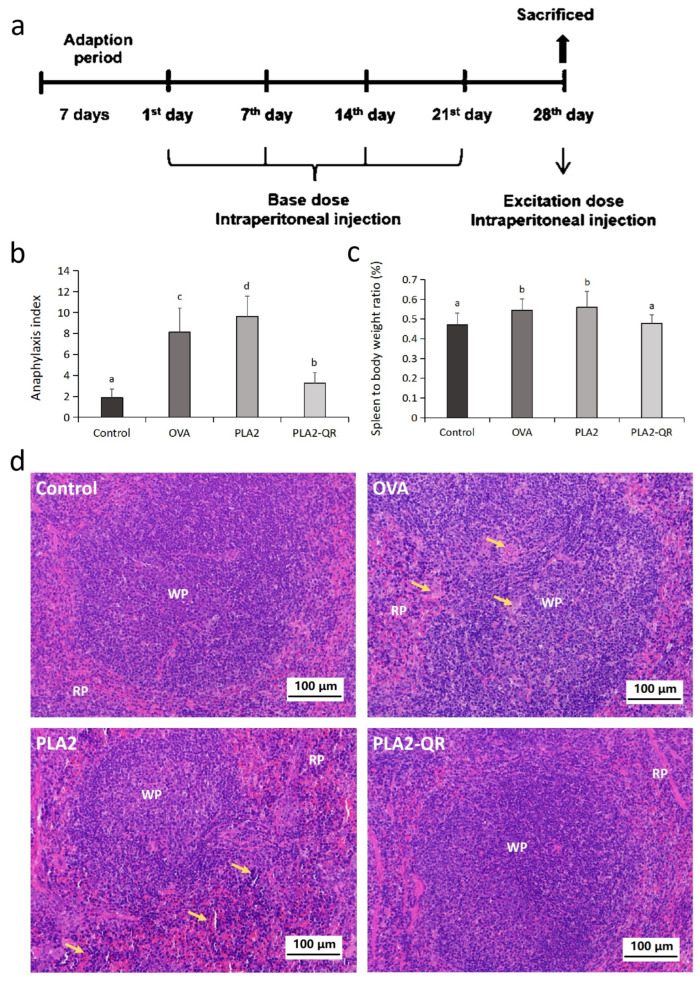
(**a**) BALB/c mouse treatment administration schedule. (**b**) Anaphylaxis index of BALB/c mice in each group (*n* = 8). (**c**) Spleen-to-body weight ratio of BALB/c mice in each group (*n* = 8). Different letters above the bars indicate significant differences (*p* < 0.05) based on Tukey’s post hoc multiple comparison test. (**d**) Histopathological changes in mouse spleen among different treatment groups stained with hematoxylin-eosin (HE). Arrows indicate inflammatory cell infiltration, RP denotes the red pulp, and WP denotes the white pulp.

**Figure 5:**
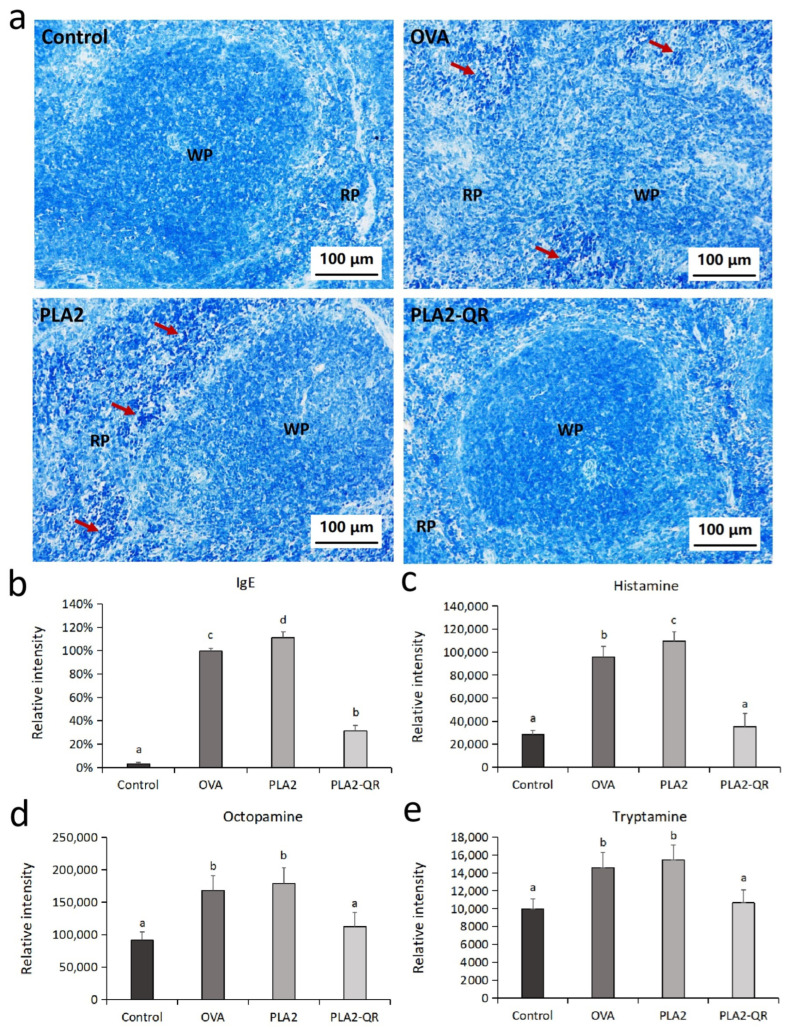
(**a**) Histopathological changes in mouse spleen among different treatment groups stained with toluidine blue (TB). Arrows indicate inflammatory cell infiltration, RP denotes the red pulp, and WP denotes the white pulp. Changes in levels of allergic indicators, namely (**b**) IgE antibody, (**c**) histamine, (**d**) octopamine, and (**e**) tryptamine in mice from each treatment group (*n* = 8). Different letters above the bars indicate significant differences (*p* < 0.05) based on Tukey’s post hoc multiple comparison test.

To evaluate mitigation of PLA2 allergenicity by QR modification, we compared native and QR-modified PLA2 allergenic potential through murine experiments. Treatment schedule is in Figure 4a. The findings suggest that both the OVA and PLA2 groups conferred notable anaphylactic responses, manifested by heightened incidences of scratching and sneezing, along with an elevated spleen-to-body weight ratio compared to the control and the PLA2-QR administration groups (Figure 4b,c). These observations were further supported by the histopathological results (Figure 4d and Figure 5a). In the control and PLA2-QR groups, the spleen exhibited an intact structure with regular morphology and a clear demarcation between the white pulp (WP) and red pulp (RP). The interstitial arrangement was tightly organized. However, in the OVA and PLA2 groups, there was an indistinct boundary between the WP and RP along with a loose interstitial arrangement in the RP region. Lymphocyte and mastocyte aggregates were observed. The observed pathological spleen phenotypes suggest a pronounced immune response in the group of mice with allergies. Moreover, serum IgE antibodies and biogenic amines indicate anaphylaxis [40,41]. IgE, histamine, octopamine, and tryptamine levels were significantly elevated in OVA and PLA2 groups versus controls (Figure 5b–e), indicating PLA2-induced allergy and increased serum IgE/bioamines. However, these levels were considerably lower in the PLA2-QR group than those observed in the PLA2 group. It indicates that the application of QR modification effectively mitigated the allergenicity of PLA2, thereby leading to a reduction in allergic responses as well as serum IgE and bioamine levels in mice. The murine sensitization models quantitatively validated the immunological consequences of structural modifications, particularly IgE reactivity and allergic responses suppression, which also strongly demonstrated the feasibility of QR in detoxification of food allergens in actual animal bodies.

## 4. Conclusions

In summary, this study resolves two critical scientific challenges in insect protein utilization: (1) the molecular mechanism underlying polyphenol-mediated allergenicity reduction, and (2) the precise targeting strategy for food allergen epitope engineering. Through an integrative approach combining computational biology and experimental validation, we established that quercetin induces site-specific covalent modification at PLA2’s Cys37 residue. This structural intervention disrupts the conformational stability of key antigenic epitopes. This research result will provide new insights for food and nutrition researchers to efficiently remove protein allergenicity and improve product safety. Crucially, our murine sensitization models demonstrated a significant decrease in IgE reactivity and allergic responses post-modification. These findings decipher the fundamental principle of phytochemical–protein interaction in allergen detoxification and bridge the critical knowledge gap between phytochemical modification and food allergen. Meanwhile, a technological paradigm for precision modification of food allergens has been established, which will contribute to the efficient detoxification and safety evaluation of complex biological matrix proteins in the future, paving the way for the development of insect-derived proteins in global food systems. Although this method shows great potential for advancing sustainable protein development, it requires further evaluation of how covalent modifications affect nutrient retention and functional properties at the matrix level. Future research should focus on these extended effects to better assess its practical feasibility.

## Figures and Tables

**Table 1:** The information of representative peptides for PLA2 and PLA2-QR conjugate after trypsin enzymatic digestion.

No.	Peptides	PTM	Mass	Mass Error (ppm)	*m*/*z*	Retention Time (min)	*z*	Assignment
1	CLHYTVDKSKPK	c	1474.7603	0.6	738.3878	7.61	2	Shared
2	CYKLEHPVTGCGER	c c	1704.7712	0.8	853.3936	15.79	2	Shared
3	FYDCLKNSADTISSYFVGK	c	2214.0303	0.8	739.0179	66.78	3	Shared
4	IIYPGTLWCGHGNK	c	1614.7976	0.3	539.2733	40.14	3	Shared
5	LEHPVTGCGERTEGR	c	1696.7950	0.5	425.2062	8.33	4	Shared
6	LSCDCDDKFYDCLK	c c c	1837.7321	0.1	919.8734	37.36	2	Shared
7	MYFNLIDTK	o	1159.5583	0.9	580.7870	45.13	2	Shared
8	NSADTISSYFVGKMYFNLIDTK	o	2529.2097	0.6	844.0777	76.62	3	Shared
9	SSGPNELGR		915.4410	1.6	458.7285	8.83	2	Shared
10	VYQWFDLRK		1253.6556	0.8	627.8356	40.87	2	Shared
11	THDMCPDVMSAGESK	c o	1679.6589	0.0	840.8367	17.67	2	PLA2
12	THDMCPDVMSAGESK	o c	1679.6589	0.3	840.8370	19.70	2	PLA2
13	THDMCPDVMSAGESK	o c o	1695.6538	1.0	848.8350	10.32	2	PLA2
14	THDMCPDVMSAGESK	o q o	1940.6324	3.4	971.3268	13.56	2	PLA2-QR

Note: PTM refers to post-translational modifications, which encompass c: carbamidomethylation; o: oxidation; q: covalent conjunction of quercetin.

## Data Availability

The original contributions presented in the study are included in the article/Supplementary Materials, further inquiries can be directed to the corresponding author.

## Supplementary Materials

The following supporting information can be downloaded at: https://www.mdpi.com/article/10.3390/nu17172872/s1, Table S1. Elution gradient procedure for the detection of bioamines; Table S2. The mass spectrometry parameters for the detection of bioamines; Table S3. The optimized ion parameters for the detection of bioamines; Table S4. Molecular docking analysis of five polyphenols binding with PLA2; Video S1: The dynamic structure of PLA2; Video S2: The dynamic structure of PLA2-QR.
