# Supplementary material for: Detoxification of Insect-Derived Allergen PLA2 via Quercetin Modification: Molecular Simulation and Animal Validation
Source: Nutrients. 2025 Sep 4;17(17):2872. doi: 10.3390/nu17172872 (PMC12430299; doi:10.3390/nu17172872)
Supplement: Supplementary file 1 [file nutrients-17-02872-s001.zip › Supplementary materials.pdf]

Supplementary

Table S1. Elution gradient procedure for the detection of bioamines.

Time (min)	A%	B%	Flow Rate (mL/min)
0	99	1	0.3
2	99	1	0.3
6	85	15	0.3
6.5	1	99	0.3
9	1	99	0.3
9.1	99	1	0.3

Table S2. The mass spectrometry parameters for the detection of bioamines.

Parameters	Condition
Ion source temperature (°C)	250
Drying gas flow rate (L/min)	7
Nebulizer pressure (psi)	30
Sheath gas temperature (°C)	325
Sheath gas flow rate (L/min)	11

Table S3. The optimized ion parameters for the detection of bioamines.

Compound	Precursor ion	Product ion	Collision energy (V)	Fragmentor (V)
Histamine	112.1	95.0*	15	80
		68.0	25	
Tryptamine	161.1	144.0	10	70
		117.0*	30	
Octopamine	154.1	136.0	4	60
		91.0*	22	

“*” means this product ion was used for quantification.

Table S4. Molecular docking analysis of five polyphenols binding with PLA2.

Complex	Binding energy	Binding sites	Conformation
PLA2+CA	-4.98	HIS34, ASP35, GLY10	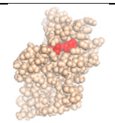
PLA2+CC	-5.83	HIS34, ASP35	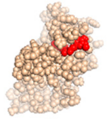
PLA2+EGCG	-6.42	HIS34, ASP35, GLY10	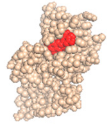
PLA2+GA	-3.67	ILE1, HIS11, ASN13	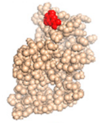
PLA2+QR	-6.49	HIS34, ASP35, THR57	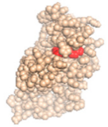

Abbreviations: CA, chlorogenic acid; CC, curcumin; EGCG, epigallocatechin gallate; GA, gallic acid; QR, quercetin.
